# Iatrogenic ulnar nerve injury after pin fixation and after antegrade nailing of supracondylar humeral fractures in children

**DOI:** 10.3109/17453674.2011.623574

**Published:** 2011-11-24

**Authors:** Robert Eberl, Christian Eder, Elisabeth Smolle, Annelie M Weinberg, Michael E Hoellwarth, Georg Singer

**Affiliations:** Department of Pediatric and Adolescent Surgery, Medical University of Graz, Graz, Austria

## Abstract

**Background and purpose:**

Ulnar nerve injury may occur after pinning of supracondylar fractures in children. We describe the outcome and compare the rates of iatrogenic injuries to the ulnar nerve in a consecutive series of displaced supracondylar humeral fractures in children treated with either crossed pinning or antegrade nailing.

**Methods:**

Medical charts of all children sustaining this fracture treated at our department between 1994 and 2009 were retrospectively reviewed regarding the mode of treatment, demographic data including age and sex, the time until implant removal, the outcome, and the rate of ulnar nerve injuries.

**Results:**

503 children (55% boys) with an average age of 6.5 years sustained a type-II, type-III, or type-IV supracondylar fracture. Of those, 440 children were included in the study. Antegrade nailing was performed in 264 (60%) of the children, and the others were treated with crossed pins. Iatrogenic ulnar nerve injury occurred in 0.4% of the children treated with antegrade nailing and in 15% of the children treated with crossed pinning. After median 3 (1.6–12) years of follow-up, the clinical outcome was good and similar between the 2 groups.

**Interpretation:**

Intramedullary antegrade nailing of displaced supracondylar humeral fractures can be considered an adequate and safe alternative to the widely performed crossed K-wire fixation. The risk of iatrogenic nerve injury after antegrade nailing is small compared to that after crossed pinning.

In children, supracondylar fractures are the most common type of fracture of the elbow region (Omid et al. 2008). Boys usually have a higher incidence of this type of fracture but some recent reports in the literature describe rising rates in girls (Cheng et al. 2001). Most of the patients are 5–7 years old (Davis et al. 2000, Omid et al. 2008, Zamzam and Bakarman 2009). At this age, the potential for fracture remodeling decreases; therefore, malreduction may lead to persistent deformity (Wessel et al. 2003).

In displaced fractures, the most common operative treatment is closed reduction and pin fixation. Different techniques have been reported, but crossed pinning with postoperative immobilization is the preferred technique (Brauer et al. 2007, Kocher et al. 2007, Zamzam and Bakarman 2009). Iatrogenic injury to the ulnar nerve has been described in up to 20% of the cases treated with crossed pinning (Lyons et al. 1998). In addition, radial pinning may damage the radial and anterior interosseous nerve (Brauer et al. 2007, Kocher et al. 2007, Omid et al. 2008).

In 1990, a technique with antegrade nailing for supracondylar fractures was first described by Prevot et al. (1990). Schaffer et al. (2007) and Weinberg et al. (2003) treated 60 and 50 children with this technique and reported no iatrogenic injuries to the ulnar nerve.

We determined the outcome and compared the rates of iatrogenic injuries to the ulnar nerve in a consecutive series of displaced supracondylar humeral fractures in children treated with either crossed pinning or antegrade nailing.

## Patients and methods

We retrospectively analyzed the medical charts of all children who had sustained a supracondylar humeral fracture and who were treated at our department between 1994 and 2009. This included age and sex, type of fracture and treatment, the time until implant removal, the outcome, and the rate of ulnar nerve injuries.

Fractures were classified according to the Gartland system as modified by Leitch et al. (Gartland 1959, Leitch et al. 2006). Type-I fractures are undisplaced or minimally displaced (< 2 mm). Type-II fractures are characterized by fracture displacement of more than 2 mm and intact posterior cortex. Type-III injuries are displaced fractures without cortical contact, and type-IV injuries are multidirectionally unstable fractures.

Type-I fractures were treated with cuff and collar immobilization. Type II injuries were treated either nonoperatively or operatively. Type-III and type-IV injuries were treated operatively.

Exclusion criteria were primarily nonoperatively treated fractures, open fractures, fractures that required open reduction, and patients with neurological abnormalities that were found at the time of presentation.

503 children (55% boys) with an average age of 6.5 (0.5–16) years sustained a type-II, type-III, or type-IV supracondylar fracture. 440 children fulfilled the inclusion criteria and were included in the study. The vast majority of these were extension-type fractures (97%, n = 427).

All patients included were treated with closed reduction and the fracture was either stabilized with percutaneous crossed pins or antegrade nailing, depending on the surgeons' preference. The method of treatment was not a matter of fracture instability.

Fracture reduction was performed in the operating room under general anesthesia and image intensifier control. The maneuver for reduction was initial traction in an extended position of the elbow joint, followed by flexion and dorsal pressure with the thumb onto the distal fragment in extension-type fractures and simultaneously pronating the forearm. Proper fracture reduction with flexed elbow joint was evaluated in a position of 90° external rotation, in the anteroposterior view, and in 90° internal rotation.

Crossed K-wires (1.6–2 mm) were inserted percutaneously and either bent outside the skin or cut below the level of the skin. An above-elbow cast was applied for 5–6 weeks. Exploration of the ulnar nerve was not performed. Early implant removal (in cases of irritation of the ulnar nerve) was not performed. Implant removal was performed together with cast removal in cases of K-wires being bent outside the skin. Children with K-wires cut below the level of the skin had the implant removed on a day-surgery basis 3 months after injury.

**Figure 1.**
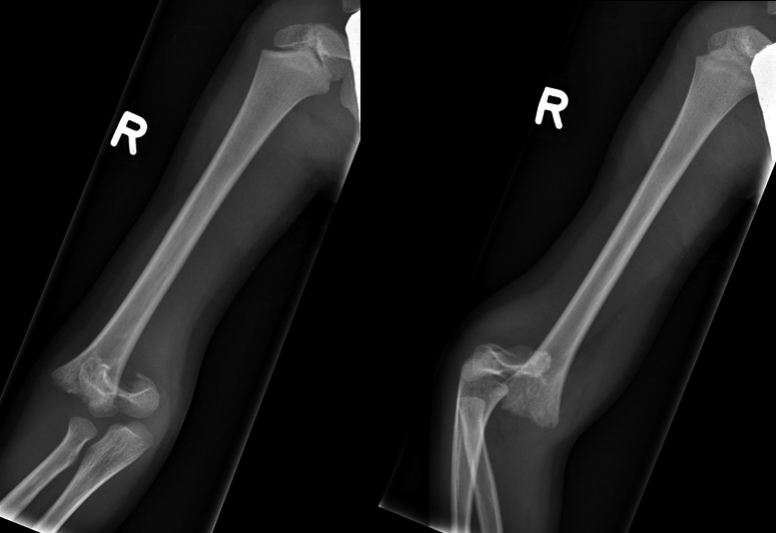
An isolated type-IV supracondylar humeral fracture in a 5-year-old boy.

**Figure 2.**
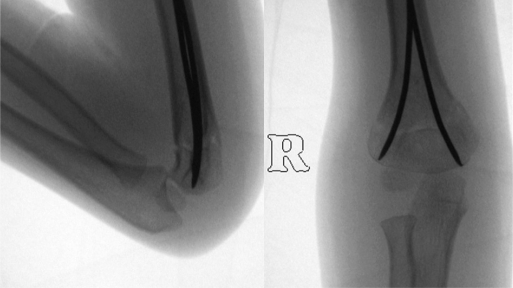
After closed reduction, antegrade nailing was performed using 2 nails. The implants spread correctly from the distal diaphysis into the radial and ulnar column. Immobilization was not required; fracture stability was high.

**Figure 3.**
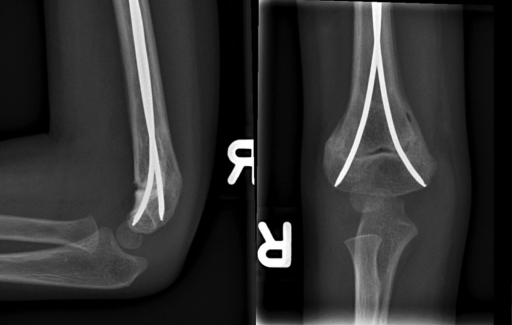
Regular follow-up 2 months after the injury, with free range of motion and normal axis of the elbow joint.

Antegrade nailing was performed with 2 K-wires (1.6–2 mm) inserted from a skin incision located 1 cm distal to the tuberosity of the deltoid muscle. The humeral cortex was opened with an awl. The K-wires were bent first and the tip was blunted to ease the insertion. The implants were positioned into the radial and ulnar column of the distal humerus (Figures 1–3). Highly unstable fractures with a high grade of instability were stabilized with a third nail. Immobilization with a cast was not performed. In cases of heavy, painful swelling of the soft tissues, a splint was used for 7–10 days. Implants were removed after 2–4 months on a day-surgery basis.

A neurological examination was done within 24 h of operative intervention. Iatrogenic injury to the ulnar nerve was treated with physiotherapy and an orthotic device during the night in cases of sensomotoric affection.

The functional outcome was graded from excellent to poor according to the Linscheid-Wheeler score (Linscheid and Wheeler 1965).

### Statistics

For comparison of groups, chi-square test was used and p-values < 0.05 were considered statistically significant.

## Results

Antegrade nailing was performed in 60% of the fractures (264 of the 440 patients) and treatment with crossed pins was performed in 40% of the fractures. The distribution of the Gartland types was similar in both groups (Table).

**Table:** 

	Antegrade nailing (n = 264)	Crossed pinning (n = 176)
Gartland type II	97 (37%)	58 (33%)
Gartland type III	125 (47%)	91 (52%)
Gartland type IV	42 (16%)	27 (15%)

Revision surgery was required in 4 patients following antegrade nailing (4/264, 2%) and in 3 patients following crossed pinning (3/176, 2%). The reasons for revision surgery were incomplete reduction with remaining displacement of the distal fragment in the 3 patients with crossed pinning, and incorrect implant placement in the 4 patients with antegrade nailing.

Iatrogenic injury to the ulnar nerve was found in 6% of the children (28/440). In the group with crossed pinning, the rate of ulnar nerve injury was 15% (27/176) (10 with sensory affection and 17 with sensomotoric affection) whereas the rate of ulnar nerve injury in the group with antegrade nailing was 0.4% (1/264) (sensory affection) (p < 0.001). Total recovery of the nerve occurred in all children after mean 9.3 (6–36) weeks.

Follow-up examinations were performed after mean 3.2 (1.6–12) years. All patients had normal motion. None had more than 10° deviation of the elbow axis relative to the unaffected side. According to the Linscheid Wheeler score, the results were good in 9% of the patients after antegrade nailing (n = 23) and excellent in 91% (n = 241). The results were similar after crossed pinning: good in 8% of the patients (n = 14) and excellent in 92% (n = 162).

## Discussion

The most common technique for operative fixation of displaced supracondylar fractures is percutaneous pinning. Different configurations of pinning have been proposed. Crossed pinning with radial and ulnar entry points is a widely used method (Babal et al. 2010). An alternative, based on the same principle, is lateral cross-wiring—also known as Dorgan's technique (Shannon et al. 2004). Skaggs et al. (2004) have described a series of supracondylar fractures with parallel radial stabilization using 2 or 3 pins. While elastic stable intramedullary nailing (ESIN) has become the gold standard for treatment of long bone fractures of the upper and lower limb (Schalamon and Petnehazy 2009), there are few reports on use of the ESIN technique for treatment of supracondylar fractures (Prevot et al. 1990, Weinberg et al. 2003, Schaffer et al. 2007).

Prevention of ulnar nerve injuries is essential for safe supracondylar fracture management. Due to the immature joint and to the posttraumatic swelling, clear anatomic landmarks are often missing and palpation of the ulnar epicondyle or ulnar nerve may not be possible. Moreover, the pin rarely directly impales the nerve but commonly restricts the nerve within the cubital tunnel by tethering adjacent soft tissue (Rasool 1998, Shannon et al. 2004, Eidelman et al. 2007, Kocher et al. 2007). A routine surgical exploration of the nerve is not recommended, but an incision over the medial epicondyle may to be useful in order to detect the ulnar groove and facilitate correct pin placement (Yen and Kocher 2008). Nevertheless, damage to the ulnar nerve is a well-known complication and affects between 2% and 20% of patients treated with crossed fixation (Lyons et al. 1998). We can confirm the high rate of ulnar nerve injuries after treatment with crossed pinning. By contrast, the rate of ulnar nerve injuries using ESIN was 0.4%. This patient suffered a displaced fracture with a very short distal fragment. During fracture fixation, the descending ulnar nail perforated the distal corticalis and injured the nerve. 2 studies have not found any ulnar nerve injuries using the ESIN technique in 110 patients in total (Weinberg et al. 2003, Schaffer et al. 2007). Considering the rates of ulnar nerve injuries, these results together with ours confirm the superiority of ESIN for treatment of displaced supracondylar fractures.

Crossed K-wire fixation in cases of supracondylar humeral fracture gives good mechanical stability (Weinberg et al. 2007). In an adult cadaver study, it has been shown that downward nailing results in poor rotational stability. These findings may be explained by an inadequate relationship between the bone dimensions of adult cadavers and the diameter of implants used in this study (Weinberg et al. 2007). In contrast to the biomechanical results, clinical studies have shown that there is adequate stability using antegrade nailing (Prevot et al. 1990, Weinberg et al. 2003, Schaffer et al. 2007). In the present study, downward nailing was performed in 264 patients. In highly unstable fractures, a third nail was inserted between the radial and ulnar implant. There was no need for revision surgery due to instability and loss of reduction. The clinical outome was good, and was similar in both treatment groups.

In summary, based on our results, intramedullary antegrade nailing of displaced supracondylar humeral fractures can be considered to be an adequate and safe alternative to the widely performed K-wire fixation. In addition, this method is clearly superior to crossed pinning regarding iatrogenic ulnar nerve injury.
